# Oral health-related quality of life (OHRQoL) in cardiovascular patients referring to Fatima Zahra Hospital in Sari, Iran

**DOI:** 10.1186/s12903-021-01756-0

**Published:** 2021-08-11

**Authors:** Tahereh Molania, Ali Malekzadeh Shafaroudi, Mehdi Taghavi, Hodis Ehsani, Mahmood Moosazadeh, Azam Haddadi, Negar Gholizadeh, Maede Salehi

**Affiliations:** 1 Department of Oral Medicine, Dental Research Center, Mazandaran University of Medical Sciences, Sari, Iran; 2 Faculty of Dentistry, Mazandaran University of Medical Sciences, Sari, Iran; 3 Student Research Committee, Faculty of Dentistry, Mazandaran University of Medical Sciences, Sari, Iran; 4 Department of Cardiology, Faculty of Medicine, Mazandaran University of Medical Sciences, Sari, Iran; 5 Department of Periodontology, Dental Research Center, Mazandaran University of Medical Science, Sari, Iran; 6 Gastrointestinal Cancer Research Center, Non-communicable Disease Institute, Mazandaran University of Medical Sciences, Sari, Iran; 7 Department of Endodontics, Dental Research Center, Mazandaran University of Medical Science, Sari, Iran; 8 Department of Restorative Dentistry, Faculty of Dentistry, Shahid Beheshti University of Medical Sciences, Babol, Iran

**Keywords:** Cardiovascular disease, Oral health, Quality of life, Periodontal disease, Xerostomia

## Abstract

**Background:**

Cardiovascular Disease (CVD) is one of the leading causes of mortality and morbidity and significantly impacts the health-related quality of life. Oral infections have been linked to cardiovascular diseases such as thrombosis, cardiac infarction, stroke, and peripheral vascular disease. This study aims to evaluate the effects of oral health on the quality of life in cardiovascular patients.

**Methods:**

The oral health-related quality of life was measured using the OHIP-14 questionnaire. Demographic information, questions regarding smoke consumption, wearing removable prostheses, nine questions regarding xerostomia, and the existence of other systemic diseases were asked from 240 participants with cardiovascular diseases. The DMFT index was clinically examined in each patient. Also, the Plaque, Gingival, and Sulcular Bleeding Indices were measured on the Ramfjord teeth. Data analysis was conducted using SPSS version 16. The independent *t* test, Mann–Whitney test, the variance analysis, and the Kruskal–Wallis test were used to compare variables in the present study. Also, regression models were used to eliminate the effect of confounding variables.

**Results:**

Gender variables, removable prosthesis, xerostomia, DMFT, and SBI were the main determinants of quality of life in CVD patients. The mean ADD-OHIP14 of participants in the study was calculated at 21.34 ± 17.40, and the SC-OHIP14 was 6.11 ± 5.07. The mean OHRQoL was higher in females than in males, and this difference was statistically significant. OHRQoL was significantly lower in patients wearing a removable prosthesis than in those without one. The relationship between age and xerostomia was significant in this study, and patients with xerostomia had a lower quality of life than those without xerostomia. Also, the mean DMFT index in subjects with xerostomia was 23.69 ± 7.76, which was statistically significant compared to those without xerostomia.

**Conclusion:**

Cardiovascular patients experienced a decreased OHRQoL. Prevention or treatment of these problems seems to justify improving the quality of life in these patients.

## Background

Cardiovascular Disease (CVD) is one of the major causes of death and disability globally, resulting in a significant increase in health care costs in society [1]. According to WHO, CVD accounts for 31% of the causes of death in the world and 20% in Iran [2]. In Eastern Mediterranean countries, including Iran, heart diseases are important health care and social issues, and their aspects are rapidly increasing [3]. Modern treatments, expanded and improved life expectancy lead to increased chronic diseases, followed by physical limitations and decreased life quality [1]. Traditional measurement criteria in treatment outcomes such as mortality and morbidity measurements do not reflect functional capabilities, mental status, and social interaction of individuals [1, 4]. Therefore, modern treatments focus on life quality and improving life expectancy, symptoms, and the functional status of patients, which is an important achievement in determining therapeutic benefits [5]. Life quality definitions are very diverse and are often defined as happiness, satisfaction, and a feeling of well-being [6]. Health-Related Quality of Life (HRQoL) is a multi-factorial concept in which physical and mental health measurements are conducted based on self-reported answers and is considered a method for measuring the results of therapeutic interventions in patients with cardiovascular diseases. Several studies have been undertaken on HRQoL in cardiovascular patients. For instance, Ko H.Y. et al. reported that CVD is significantly associated with impaired HRQoL [4]. Oral Health-Related Quality of life includes a part of life quality that is mainly affected by oral health; in other words, it demonstrates the effect of oral diseases on the quality of life in patients [7]. Difficulties such as xerostomia, edentulousness, soft tissue lesions, poorly adapted prostheses, pain, infections, and limitation in eating and drinking are problems caused by the lack of oral health, which simultaneously affect the overall systemic health and life quality of patients [8]. Oral health and general health cannot be considered separately, as studies have clarified the link between oral and systemic diseases such as CVD [9]. Poor oral health in CVD patients leads to increased oral debris, calculus, and periodontal diseases, which increase coronary artery diseases (CAD). Studies have also reported that topical and systemic infections can start or be related to the progression of atherosclerotic plaques [10]. Questionnaires can be used for evaluating the quality of life associated with oral health. To evaluate the effects of oral health on the life quality of patients with cardiovascular disease, the OHIP-14 questionnaire was used in the present study, which Slade first used in 1997 to evaluate seven aspects of quality of life associated with oral health [8]. The relationship between OHRQoL and different chronic diseases has been addressed in previous studies [11, 12]. Due to the lack of sufficient studies that examine the OHLQoL in CVD patients, we evaluated the importance of CVD as a chronic disease, its effects on oral health, and the lack of studies on the impact of oral health on the quality of life in CVD patients. Therefore, the present study aims to investigate the oral health-related quality of life (OHQoL) in cardiovascular patients referred to Fatima Zahra Hospital in Sari, Iran, in 2019.

## Methods

This descriptive-analytic epidemiologic study was conducted on patients with CVD referred to Fatima Zahra Hospital in Sari, Iran, in 2019. The average estimation formula was used to determine the sample size. According to a study conducted by Abedi et al., the standard deviation of the quality-of-life score was 15.57. Considering this standard deviation, with an acceptable error of 2 and a 95% confidence interval, the sample size of the present study was estimated to be 240 subjects [13]. By starting the study on 1/25/2019, Samples matching the inclusion criteria entered the study using the census method until the total sample size reached 240 subjects by 3/23/2019. In other words, it took approximately 3 months to determine the total sample size of all patients referring to Fatemeh Zahra Heart Hospital. In this period, all eligible samples had the chance to enter the study. The inclusion criteria were patients with CVD who had at least 20 remaining teeth; illiterate people and those unable to complete the questionnaire were excluded from the study.

First, all participants received an explanation about the purpose and stages of this study. Then, informed consent was obtained from all subjects. After oral approval, demographic information, including age, gender, and occupation, was recorded. Only one trained questioner was involved in completing the questionnaires in the present study. Questions about smoking, wearing removable prostheses, and nine other questions regarding xerostomia were asked from each individual. Patients who responded positively to five of nine oral dryness questions were considered patients with xerostomia [14]. Information about the presence of other possible systemic diseases was also documented.

It should be noted that the group classified as “Others” includes patients with other underlying diseases or several systemic diseases. Due to the small number of these patients in each subgroup, they were classified as a separate group named “Others.” Measurement of oral health related to the quality of life was conducted using the OHIP-14 questionnaire; the validity and reliability of the Farsi version were confirmed [14]. The Alpha’s Cronbach for each OHIP-14 domain, item, and the total score is presented in Table 1 for seven subgroups, including functional limitation, physical pain, physical discomfort, physical disability, psychological disability, social disability, and handicap. Two different methods were used to evaluate the responses: A.D.D. (additive) method in which the test options were scored as follows: never = 0, seldom = 1, sometimes = 2, almost often = 3, and in the majority of cases = 4. The ADD-OHIP-14 rating has a variable between 0 and 56; the lower the score, the better the patient's life quality. In another method, Simple Count (SC), the score 0 was allocated for the options ‘never’ and ‘seldom’, and score one was earmarked for the options ‘sometimes’, and ‘almost often’, which was found to be the case in the majority of answers. This method was considered because some people may not have understood the actual difference between the questionnaire options. Using this method, the score SC-OHIP-14 ranged from 0 to 14. A lower score, once again, pointed to a higher quality of life in the patients [14].

**Table 1:** The Alpha’s Cronbach for each OHIP-14 domain and total score

Domain		Cronbach’s alpha	Item
1	Functional limitation	0.259	1. Trouble pronouncing words
			2. Sense of taste worse
2	Physical pain	0.552	3. Painful aching in mouth
			4. Uncomfortable to eat
3	Psychological discomfort	0.927	5. Self-conscious
			6. Felt tense
4	Physical disability	0.965	7. Unsatisfactory diet
			8. Had to interrupt meals
5	Psychological disability	0.909	9. Difficult to relax
			10. Embarrassed
6	Social disability	0.925	11. Irritability with others
			12. Difficulty doing usual jobs
7	Handicap	0.815	13. Felt life less satisfying
			14. Totally unable to function
Total		0.953	

The patients were clinically examined, and their DMFT and Plaque Indices (PI) were recorded. Each patient’s Gingival Index (GI) was measured according to Loe and Silness method, and the Sulcus Bleeding Index (SBI) was examined on each individual’s Ramfjord teeth (teeth number 3, 9 12, 19, 25, 28). If one of the mentioned teeth was absent, the adjacent teeth were examined. Data were entered into the SPSS version 16 software. The Shapiro–Wilk test assessed the normality of quantitative variables. All variables were described using mean, standard deviation, and frequency. To compare quantitative variables between two groups, the independent *t* test or Mann–Whitney test, and the comparison of more than two groups, the analysis of variance or the Kruskal–Wallis test was used. The Chi-square test was used to examine the classified variables. In addition, Spearman’s correlation coefficient was used to investigate the relationship between the patients’ quality of life and quantitative variables such as age, PI, GI, SBI, and DMFT. Next, the Multivariate logistic regression test was used to evaluate the factors related to xerostomia in patients with cardiovascular disorders. Eventually, the Multivariate linear regression test was also used to evaluate the factors associated with the quality of life score in patients with cardiovascular disease. The significance level was considered as *P* value < 0.05.

## Results

Of the 240 patients who participated in this study, 148 (61.7%) were male, and 92 (38.3%) were female, all suffering from CVD. The mean age of the subjects was 59.34 ± 18 (29–88 years). 68% of the patients in either one or both of their jaws used a removable partial denture. A history of alcohol consumption was reported in 14 patients (5.8%), 48 (20%) had a history of smoking, 107 (44.6%) only suffered from CAD, and 133 (55.4%) had other systemic diseases. 33 (13.8%) patients had diabetes, 15 (6.2%) had high blood pressure, 16 (6.7%) had diabetes and high blood pressure, and 9 (3.8%) had kidney diseases and other illnesses. There were thyroid problems, neurological diseases, asthma, gout, prostate problems, Parkinson’s disease, thalassemia, and joint rheumatism. The involvement of three or more systemic diseases was documented as other groups in the results. According to the present study criteria, xerostomia was positive in 102 (42.5%) patients and negative in 138 (57.5%). The average xerostomia was calculated at 3.37 ± 3.5, and the average PI was 1.98 ± 0.62. The average GI was 1.68 ± 0.75, and the average SBI was 0.78 ± 0.39. The mean ADD-OHIP14 of participants in the study was calculated at 21.34 ± 17.40, and the SC-OHIP14 was 6.11 ± 5.07. The mean DMFT was calculated to be 20.96 ± 8.26. (Table 2).

**Table 2:** Mean ± SD, median, and range of study variables

Variables	Number (n)	Mean ± SD	Median	Range
Age	240	59.34 ± 1.18	59	29–88
PI	240	1.98 ± 0.62	2	0/25–3
GI	240	1.68 ± 0.75	2	0–3
SBI	240	0.78 ± 0.39	1	0–1
Xerostomia	240	3.37 ± 3.15	3	0–9
ADD-OHIP14	240	21.34 ± 17.40	19	0–56
SC-OHIP14	240	6.11 ± 5.07	5	0–14
DMFT	240	20.96 ± 8.26	22	2–32

To investigate xerostomia and its relationship with quantitative variables, the Mann–Whitney test was used. The following results were obtained: The mean age of patients with xerostomia was 62.12 ± 11.9, the relationship between age and xerostomia was significant in this study (*P* value = 0.002). The plaque index was higher in patients with xerostomia than in patients without xerostomia, but this relationship was not statistically significant. The SBI was 0.8 ± 0.37 in patients with xerostomia and 0.76 ± 0.4 in patients without xerostomia, and this relationship was not statistically significant. The mean of life quality was lower in patients with xerostomia than in those without xerostomia (*P* value = 0.000). Finally, the mean DMFT index in subjects with xerostomia was 23.69 ± 7.76, statistically significant (*P* value = 0.000) than those without xerostomia (Table 3).

**Table 3:** The relationship between xerostomia and measured quantitative variables

Variables	Patients with xerostomia		Patients without xerostomia		*P* value
	Mean ± S.D	Number (n)	Mean ± S.D	Number (n)	
Age	62.12 ± 11.90	102	57.28 ± 11.35	138	0.002*
PI	2.01 ± 0.56	102	1.97 ± 0.66	138	0.879
GI	1.63 ± 0.72	102	1.71 ± 0.77	138	0.481
SBI	0.8 ± 0.37	102	0.76 ± 0/40	138	0.399
OHRQOL	26.32 ± 16.93	102	17/65 ± 16.87	138	< 0.001*
DMFT	23.69 ± 7.76	102	18.94 ± 8.06	138	< 0.001*

^*^P value < 0.05

The Chi-square test was used to investigate the qualitative variables in this study and their relationship with the dependent variable of xerostomia. The results are as follows: Xerostomia was observed in 55 males (37.2%) and 47 females (51.5%), which showed a significant association between gender and xerostomia (*P* value = 0.034). In 35 patients (51.5%), Xerostomia was observed with a removable partial denture and 67 (39%) patients without a removable partial denture. The relationship between using removable partial dentures and xerostomia was not statistically significant. Xerostomia was positive in 6 of 14 (42.9%) patients with a previous alcohol consumption history. The relation between the smoking history of patients and xerostomia was not significant in this study; however, xerostomia was seen in 20 (41.7%) smokers. Xerostomia was observed in 38 patients (35.5%) with CVD and 64 (48.1%) patients with CVD who also suffered from other systemic diseases. Despite the higher frequency, this relationship was not statistically significant. In the study of factors related to xerostomia, the variables of age, gender, removable partial denture, cigarette and alcohol consumption, DMFT, PI, GI, SBI, and systemic diseases were included in the regression model, which adjusted the effect of each of these variables. Related factors to xerostomia were done using a logistics regression test. The impact of the suspected confounding variables was moderated. The odds of xerostomia in women were 1.8 times greater than in men. In other words, it can be stated that the risk of xerostomia in women with CVD is 82% higher than in men after adjusting the effects of these variables, but this difference was not statistically significant. Assessing the variable of age, xerostomia increases by 0.1, which is a minimal amount for each year increase in age.

Also, in CVD patients wearing a removable partial denture, the rate of xerostomia was 0.86 times higher than cardiovascular patients without a removable partial denture. In other words, the odds of xerostomia in cardiovascular patients with a removable partial denture are 14% lower than those without one. The probability of xerostomia in people with CVD who report alcohol consumption is 1.55 times greater than that of people who did not have a history of alcohol abuse in the past, but this difference was not statistically significant. The odds of xerostomia in CVD patients with a history of smoking are also 1.05 times greater than that of patients who had not used cigarettes in the past. Again, this relationship was not statistically significant. The results showed that each unit increase in the DMFT index increased the odds of xerostomia by 1.09, which was statistically significant (*P* = 0.001). In those cardiovascular patients classified as the “Others” group in the study, the risk of xerostomia was also 2.33 times higher, which was statistically significant (*P* value = 0.017). The odds of xerostomia grow 1.39 times higher with an increase in the PI index, but this difference was not statistically significant. With an increase in the SBI, the odds of xerostomia are multiplied by 2.25, but the difference is not statistically significant. However, an increase in GI decreases the odds of xerostomia by 46%, which is statistically significant (*P* value = 0.041) (Table 4). The Hosmer–Lemeshow test was performed for the logistic regression model. The significance level of the model based on the results of the Hosmer and Lemeshow test was 0.224. Since this value is above 0.05, the null hypothesis of an acceptable explanation of the data is confirmed by the model.

**Table 4:** Factors related to xerostomia in CVD patients (multivariate logistic regression)

Variable	Odds ratio	Confidence interval 95%	*P* value	Wald test
Gender	1.79	0.92–3.48	0.089	2.899
Age	1.00	0.97–1.03	0.989	0.000
Removable partial denture	0.86	0.43–1.69	0.657	0.197
Smoking	1.05	0.45–2.47	0.903	0.015
Alcohol consumption	1.55	0.42–5.73	0.513	0.428
DMFT	1.09	1.04–1.14	0.001*	11.838
Diabetes	1.10	0.46–2.62	0.832	0.045
High blood pressure	1.49	0.44–5.02	0.519	0.415
Diabetes and high blood pressure	1.31	0.41–4.22	0.651	0.205
Kidney disorders	0.90	0.19–4.25	0.899	0.016
Others	2.33	1.11–4.91	0.026*	4.973
GI	0.54	0.30–0.97	0.041*	4.180
PI	1.39	0.68–2.84	0.366	0.816
SBI	2.25	0.84–6.03	0.107	2.602

^*^P value < 0.05

According to logistic regression analysis, DMFT, other systemic diseases, and GI were the significant determinants of xerostomia in cardiovascular patients. It should be noted that the variables which were entered in the multivariate logistics regression model of factors affecting xerostomia showed that these variables predicted 19.6% of the outcomes of xerostomia in patients with cardiovascular disease. The Spearman Correlation Test was used to examine the other dependent variable of this study, the oral health relating to the quality of life, with quantitative independent variables. The relationship between age, PI, and GI with OHRQoL was not statistically significant, but the relationship between SBI and OHRQoL was statistically significant (*P* value = 0.012). The relationship between the DMFT index and quality of life was statistically significant (*P* value = 0.000) (Table 5).

**Table 5:** The relationship between OHRQoL and quantitative variables studied

Quantitative variables	r (spearman correlation coefficient)	*P* value
Age	0.07	0.293
PI	0.06	0.368
GI	0.11	0.093
SBI	0.16	0.012*
DMFT	0.24	< 0.001*

^*^P value < 0.05

The following results were obtained assessing the relationship between qualitative variables and oral health-related quality of life. The mean OHRQOL in women was higher than in men, and this difference was statistically significant (*P* value = 0.026). OHRQoL was lower in patients wearing a removable partial denture than in those without one, and this difference was statistically significant (*P* value = 0.009). The average score of OHRQoL in people with alcohol abuse was higher than in those who did not consume alcohol, but this difference was not statistically significant. The mean OHRQOL score in subjects with a smoking history was higher than in non-smokers, and this relationship was not statistically significant. On average, the life quality score in patients who also suffered from other systemic diseases was lower than those who only suffered from CVD. Still, this difference was not statistically significant (Table 6).

**Table 6:** The relationship between OHRQoL and qualitative variables

Qualitative variables	Number (n)	Mean ± SD	*P* value
Gender			
Male	148	19.66 ± 17.51	0.026*
Female	92	24.04 ± 16.96	
Removable partial denture			
Uses	68	16.76 ± 16.57	0.009*
Does not use	172	23.15 ± 17.44	
Alcohol consumption			
Positive	14	24.57 ± 18.96	0.634
Negative	226	21.14 ± 17.33	
Smoking			
Positive	48	25.39 ± 18.82	0.109
Negative	192	20.32 ± 16.93	
Co-morbidity with other systemic diseases			
Positive	133	20.95 ± 17.36	0.879
Negative	107	21.82 ± 17.52	

^*^P value < 0.05

Assessing the factors related to OHRQoL and using multivariate linear regression, the effect of age, gender, removable partial denture, smoking and alcohol consumption, DMFT, PI, GI, SBI, and a history of systemic diseases were modified. According to the multivariate linear regression analysis results, the quality of life in women with CVD was 0.19 units higher than in men; women had a lower quality of life than men. For each unit increase in age, the OHRQoL score is reduced by 0.04 units. The quality-of-life scores in patients with a removable partial denture were 0.32 times lower than those without a removable partial denture. This difference was statistically significant (*P* value = 0.000). The OHRQoL score in patients with a history of alcohol consumption increased by 0.03, and in those with a history of smoking increased by 0.01 units. Finally, the OHRQoL score in patients with xerostomia increased by 0.15 units. In other words, patients with xerostomia had a worse quality of life than those without xerostomia. This difference was statistically significant (*P* value = 0.012). The OHRQoL score for an increase in each unit of DMFT also increased by 0.35 units, and this difference was statistically significant (*P* value = 0.000). The OHRQoL score in patients with systemic diseases decreased, but it was not statistically significant. The OHRQoL score increased by 0.03 times with an increase in the GI score and decreased by 0.13 times by increasing the PI score, none of which were statistically significant. Each unit increase in the SBI score increased by 0.29 times in the OHRQoL score, and this difference was statistically significant (*P* value = 0.008) (Table 7).

**Table 7:** Factors related to the OHRQoL in CVD patients (multivariate linear regression)

Variables	Beta	*P* value
Gender	0.19	0.004*
Age	− 0.04	0.592
Removable partial denture	− 0.32	< 0.001*
Smoking	0.10	0.142
Alcohol consumption	0.03	0.631
Xerostomia	0.15	0.012*
DMFT	0.35	< 0.001*
Diabetes	− 0.04	0.474
Blood pressure	− 0.00	0.955
Diabetes and blood pressure	− 0.08	0.168
Kidney disorders	− 0.04	0.436
Others	− 0.01	0.770
GI	0.03	0.728
PI	− 0.13	0.158
SBI	0.21	0.008*

^*^P value < 0.05

Generally, the linear regression model results showed that the variables of gender, parietal prosthesis, xerostomia, SBI, and DMFT are the main determinants of OHRQoL in patients with cardiovascular disease. It should be noted that the variables included in the multivariate linear regression model account for 21% of changes in the oral health-related quality of life in cardiovascular patients.

## Discussion

Many studies have evaluated the effects of oral problems on the life quality of patients with common systemic diseases [15, 16]. CVD patients showed oral complications that seem to be related to the life quality associated with their oral health. Based on the findings of this study, the average oral health-related quality of life was 21.34 ± 17.40. Assessing factors related to life quality, the effects of variables such as age, gender, removable partial denture, smoking and alcohol consumption, DMFT index, systemic diseases, PI, GI, and SBI. were modified using multivariate linear regression analysis. It was concluded that variables of gender, removable partial denture, xerostomia, DMFT, and SBI. were the main determinants of quality of life in CVD patients. According to the results, women had a lower quality of life than men. Patients with a removable partial denture had a better OHRQoL than those without one. Patients with xerostomia and a higher DMFT index had a lower quality of life. The present study is the first study on oral health-related quality of life in cardiovascular patients that addresses the similarities and differences. In Motalebnejad et al. study [14] regarding the effects of diabetes on OHRQoL, women showed a lower quality of life than men. This could be related to the age and post-menopausal status of women. Menopause effectively increases stress levels in women. Studies have shown that psychological stress increases in women before and after menopause, affecting their OHRQoL [1]. In a study by Jiange et al., women showed a lower quality of life than men, related to a more severe psychological status in women than men [17]. Martinelli et al. suggested that being a male decreases the chance of lower scores in physical and psychological functions and observed a similar result in their study. The main reason for the decrease in the quality of life in women compared to men was not mentioned. Still, the possible reasons for this decrease in women were lower self-esteem, hard work, and perhaps workplace problems [18]. The use of a dental prosthesis leads to improvements in the chewing and beauty of the patient, which seems to improve the oral health-related quality of life in these cases. Xerostomia can lead to several oral issues, including increased dental caries, halitosis (bad breath), burning sensation, dental plaque accumulation, atrophy, mucositis, ulcers, and opportunistic infections (bacterial, fungal, and viral), and gingival diseases [19, 20]. According to the results of this study, the OHRQoL was lower in patients with xerostomia who had a higher DMFT index, which is reasonable considering the effects of xerostomia on oral health. Jellema et al. assessed the effects of radiation-induced xerostomia on the life quality of patients with head and neck cancer, which concluded that there were significant negative effects between these parameters in individuals. They emphasized the importance of preventing xerostomia from preventing further complications and improving the quality of life in patients [19]. Another study by Henson et al. assessed the effects of oral dryness on the quality of life in patients receiving parotid gland radiotherapy. This study was consistent with the present study, suggesting that the worst quality of life-related to xerostomia occurs during radiation therapy. A few months after radiotherapy, subsequent to improving salivary gland function and increasing the salivary flow, the quality of life in patients increased significantly [20]. A study by Van Rij used a more recent treatment approach instead of conventional radiotherapy, which led to less damage to the salivary glands. Therefore, it reduced the gland dysfunction resulting in a significant improvement in the quality of life-related to xerostomia in patients [21]. Bakhtiar et al. [22] reported a positive correlation between the DMFT index and quality of life in Iranian adolescents. Tubert-Jeannin et al. [23] also found a similar relationship between decayed teeth and oral health-related to the quality-of-life index, which is consistent with the results of the present study. Biazevic et al. [24] also assessed the relationship between oral health and the quality of life, suggesting a positive and significant correlation between the highest OHIP score and the DMFT index. Mtaya et al. [25] found no significant association between the DMFT index and the OHRQoL in children, contradicting the present study results, which seems to be due to age and systemic disease condition differences of the participants in the two studies. The present study also modified the odds of xerostomia incidence by using a multivariate logistic regression test for variables such as age, gender, removable partial denture, smoking and alcohol consumption, DMFT index, systemic diseases, PI, GI, and SBI. in cardiovascular patients. It was observed that the DMFT index, GI, and systemic disease variables were the main determinants of xerostomia in the mentioned patients. The effects of xerostomia on oral health and tooth decay and oral health indices have been reported in several studies, confirming the present study results [19, 20, 26]. Several factors may lead to xerostomia, namely topical and psychological factors, and systemic diseases being the main etiology of this condition [26]. Studies have stated that the anticholinergic effects of many drugs, alcohol consumption, and radiotherapy in the head and neck region are the main causes of xerostomia [27]. Therefore, considering the advanced age of the participants in this study and the co-morbidity of other systemic diseases and CVD and simultaneous usage of multiple drugs, it seems that the relationship between systemic diseases and xerostomia in these subjects can be justified. Notably, the plaque, gingival, and sulcular bleeding indices were measured in cardiovascular patients in this study. Many studies have assessed the relationship between periodontal and cardiovascular diseases [28]. Rutger Persson's study evaluated the relationship between periodontitis and myocardial infarction and eventually suggested that the risk of CVD increases with the severity of periodontal disease [28]. Genco et al. concluded that alveolar bone loss was more pronounced in patients with diabetes mellitus and CVD [29]. In Meurman,study which explored a variety of studies on the relationship between periodontal and cardiovascular disease, it has been suggested that designing and reaching an agreement on the relationship between these two categories is difficult (30). An increase in the plaque, gingival, and sulcular bleeding indexes in cardiovascular patients increased their quality-of-life scores resulting in worse quality of life for these individuals. Also, CVD patients with xerostomia had a lower quality of life than those without xerostomia. It seems that the prevention or treatment of these problems is justified to improve the quality of life in these patients.

## Conclusion

Cardiovascular patients experienced a worse OHRQoL due to oral problems, including xerostomia and increased plaque, gingival, and sulcular bleeding indices. Therefore, prevention or treatment of these problems seems to justify improving the quality of life in these patients.

## Strengths and limitations

According to our knowledge, this is the first study that assessed OHLQoL in cardiovascular patients using the OHIP-14 questionnaire and by clinically examining oral indices such as DMFT, PI, GI, and SBI. Due to the wide range of lesions and oral problems and the impact that each of these problems can have on the OHLQol, we were only able to evaluate the effects of a few of these issues. Therefore, further studies are necessary to confirm that these results are generalizable to the entire cardiovascular patients. Different medications are prescribed for cardiovascular patients that may affect OHRQoL in these patients. However, due to the wide range of drugs used in these patients and the prolongation of the results evaluating the effect of each of these drugs on the OHRQoL was not possible for the authors. This is considered as one of the limitations of the present study. We recommend further studies to evaluate the effect of medications taken by the participants on their OHRQoL. Another limitation of the present study was the lack of determination of Intra-rater reliability, which approves the consistency in ratings given by the same individual across multiple samples prior to the inception of the study.

## Data Availability

The datasets used and/or analyzed during the current study are available from the corresponding author on reasonable request.

## Abbreviations

CVD: Cardiovascular disease
DMFT: Decayed, missing, and filled teeth
PI: Plaque index
GI: Gingival index
SBI: Sulcus bleeding index
OHLQoL: Oral health related quality of life
WHO: World Health Organization
CAD: Coronary artery disease
SC: Simple count
